# Ketamine infiltration decreases the need for opioids after thyroid surgery

**DOI:** 10.12688/f1000research.127562.1

**Published:** 2023-02-23

**Authors:** Moncef Sellami, Imen Zouche, Mariam Ben Ayed, Maroua Bouhali, Khadija Ben Ayed, Salma Ktata, Boutheina Hammami, Mohamed Amine Chaabouni, Ilhem Charfeddine

**Affiliations:** 1 Faculty of Medicine of Sfax, Sfax, Tunisia; 2 University of Sfax, Sfax, Tunisia; 3 Department of Otorhinolaryngology, Head and Neck Surgery, Habib Bourguiba University Hospital, Sfax, Tunisia; 4 Department of Anesthesia, Habib Bourguiba University Hospital, Sfax, Tunisia

**Keywords:** Ketamine, thyroid surgery, wound infiltration, analgesia

## Abstract

**Background:** Postoperative pain increases the risk of postoperative complications and may predispose patients to chronic post-surgical pain. This study aims to evaluate the impact of ketamine wound infiltration versus placebo at the end of thyroid surgery on postoperative pain and analgesic requirements.

**Methods:** In this randomized controlled trial, we prospectively studied patients who underwent thyroid surgery. Patients were randomized into two groups: group S, where local infiltration was performed using 10 ml of a physiological saline solution; and group K, where 10 ml of a solution containing 2 mg/kg ketamine was infiltrated. Standardized thyroidectomies were performed in the 2 groups. Pain perception was measured using a visual analog scale (VAS) every 10 minutes in the post-anesthetic care unit (PACU) for 2 hours and thereafter every 6 hours during the first 24 hours. The opioid requirement in the PACU was evaluated. A comparison between the 2 groups was carried out.

**Results:** Postoperatively, the mean VAS was higher in group S compared to group K during all PACU stay periods and the first 24 hours. Pain scores during swallowing were significantly lower for group K in the PACU at 0, 10, and 20 minutes. The mean morphine consumption in the PACU was 0.71 mg and 0 mg respectively in group S and group K (p=0.03). The incidence of nausea and vomiting was similar in both groups.

**Conclusions:** Ketamine wound infiltration is an efficient modality to reduce postoperative opioid consumption compared to a placebo after thyroid surgery.

## Introduction

Thyroid surgery is known to be responsible for mild to moderate postoperative pain during the first 24 hours after surgery. ^1^ Postoperative pain can result in significant discomfort, delay in hospital discharge, and the development of chronic pain. ^2^ Postoperative pain control is fundamental for better recovery and quick return to daily activities. Opioids are effective for postoperative analgesia, but they cause sedation, respiratory depression, nausea, and vomiting. ^3^ The modern concept in pain management includes a multimodal approach that involves the use of systemic analgesics associated with locoregional anesthesia techniques to reduce postoperative pain and opioid requirement. ^4^^,^^5^ The locoregional anesthesia technique has mainly included bilateral superficial cervical plexus block and local wound infiltration with a local anesthetic agent. ^1^^,^^6^^,^^7^ Bilateral block of the superficial cervical plexus is an effective technique that ensures better postoperative analgesia and has been widely used in thyroid surgery. ^6^^,^^8^ Wound infiltration with a local anesthetic agent is a simple and safe procedure for reducing post-operative pain. ^6^^,^^7^ In the current literature, different anesthetic agents for thyroid surgery wound infiltration were studied, including bupivacaine, diclofenac, ropivacaine, and, more rarely, ketamine. ^9^^–^^12^

Ketamine has a high affinity for N-methyl-D-aspartate receptors; it can also bind to opioid mu and sigma receptors that provide central and peripheral analgesic effects. ^13^

The purpose of this study was to assess the impact of local wound infiltration using ketamine at the end of thyroid surgery on postoperative pain and opioid requirement.

## Methods

### Selection of patients, ethical approval and consent

Ethical approval for this study was given by the Committee for the Protection of People in Southern Tunisia (approval number 0117/18). This randomized double-blind study was achieved through collaboration between the ENT department and the anesthesia department of the university hospital, Habib Bourguiba in Sfax, Tunisia.

Patients scheduled for thyroid surgery were enrolled after having provided written informed consent, which they also gave for publication of their data, in de-identified form.

Patients aged 18 to 65 were included in this study if they had an ASA score (American Society of Anesthesiology) of I or II. Patients with unstable diabetes, an allergy to study drugs, a history of previous cervical surgery, or a history of cardiac or respiratory disease, as well as patients on long-term analgesics or corticosteroids, were not included in this study. Patients who had major complications, such as allergic reactions to anesthetic drugs and major bleeding, or patients whose surgery duration exceeded 3 hours were excluded from this study. Patients who underwent a neck dissection associated with thyroid surgery were also excluded from the study.

### The patients and randomization

The patients were informed about the anesthetic protocols and were educated about the use of the visual analog scale (VAS) to evaluate the severity of the pain. All patients in the study were blinded to the drug they received for postoperative pain.

These patients were randomized into two groups: group K represents wound infiltration using 10 ml of a solution containing 2 mg/kg of ketamine and group S (placebo group) represents wound infiltration using 10 ml of normal saline solution. The wound injection was performed at the end of the surgery after the skin suture.

### General anesthesia procedure

After 3 minutes of preoxygenation, anesthesia was induced with an injection of 3 μg/kg of Fentanyl followed by 3 mg/kg of Propofol; and with intubation using a silicone wire-reinforced tracheal tube with 0.2 mg/kg Cisatracurium. Anesthesia was maintained using isoflurane with a minimum alveolar concentration (MAC) of 1% in a 50% oxygen/air mixture. A 0.03 mg/kg Cisatracurium bolus was administered every 40 min, and a Fentanyl bolus of 0.1 μg/kg was injected whenever there was an increase of 20% of the base values in heart rate or systolic arterial blood pressure.

Before surgery, an anesthesiologist, not involved in the study, prepared an unlabeled sterile syringe using 10 ml of ketamine (2 m/kg) or 10 ml of a saline solution. After wound closure, the infiltration was performed by a blinded surgeon. The needle was introduced to a depth of 0.5 cm, an aspiration was then performed to avoid an intravascular injection followed by infiltrating the wound. The content of the syringe was used for homogenous infiltration of the subcutaneous wound sides by the surgeon.

### Analgesic protocol

Thirty minutes before the end of the surgery, all patients received 1 g of paracetamol ad then were extubated before being moved to the post-anesthetic care unit (PACU) for close monitoring for 2 hours. In the PACU, intravenous morphine titration (2 mg every 5 min) was performed until the VAS value was less than 30. All patients were admitted for at least 24 hours postoperatively in the ENT department. They all received 1 g of paracetamol systematically every 6 hours during the first 24 hours. An anesthesiologist blinded to study groups collected intraoperative and postoperative parameters.

### Evaluation criteria

The primary outcomes were to determine the intensity of the pain using VAS from 0-100 in the first 24 hours. The VAS score was assessed every 10 minutes in the PACU for 2 hours and every 6 hours during the first 24 hours after the operation in the ENT department. Nefopam was administered in the cases where VAS exceeded 30. Opioid requirements were recorded during the PACU admission period. The occurrence of side effects of opioids and ketamine was noted.

### Statistical analysis

In each group, a total number of 27 patients was required to obtain a difference on the VAS scale of 20 mm (standard deviation of 25 mm), with a power of 0.9 and an α=0.05. To allow for some margin of error in the underlying assumptions, the baseline sample size was increased by 10 % to 32 patients per group. χ ^2^ test or Fisher statistical tests were used for the analysis of qualitative variables and the Student's T test for the analysis of quantitative variables (sample size of more than 30 patients per group; normality was typically ensured by the central limit theorem). A p-value < 0.05 was considered statistically significant (one-tailed test). Data entry and statistical analysis of data were performed with the software version 20 of Statistical Package for Social Sciences (SPSS) for Windows (SPSS Inc., Chicago, IL, USA).

## Results

### Study population

**Figure 1.**
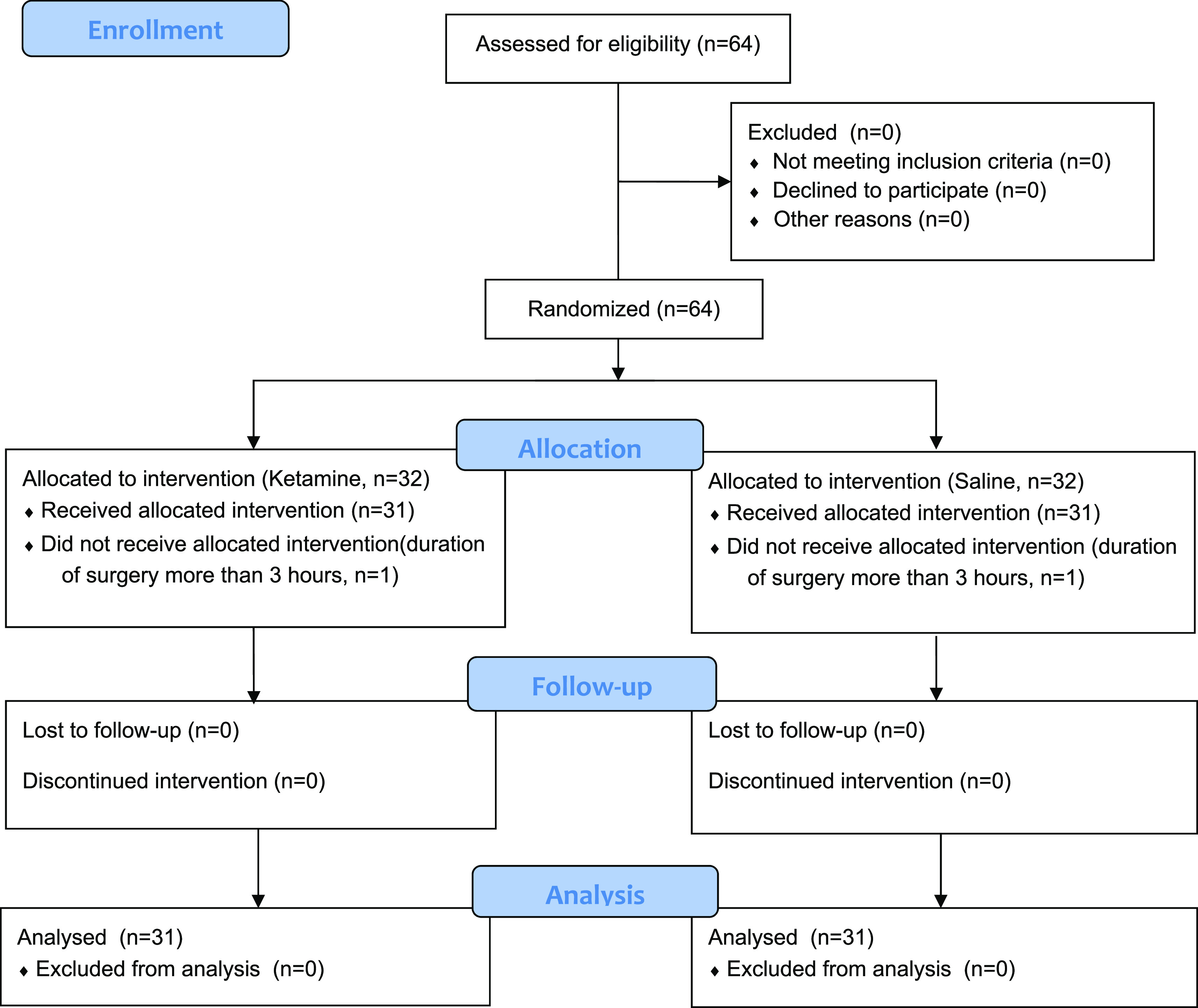
CONSORT 2010 flow chart for the patients in the study.

The study lasted for a total of 6 months from September 2018 to March 2019. Sixty-four patients were included. Two patients were withdrawn from the study for prolonged surgery (more than 3 hours). The flow of patients is represented in the CONSORT diagram of the study, which is shown in Figure 1. Demographic and intraoperative characteristics of patients are represented in Table 1.

**Table 1.** Demographic characteristics and intraoperative anesthetic characteristics of both groups.

		Group S (N=31)	Group K (N=31)	P value
**Demographic characteristics**	Age (years)	47.9±13	41.8±38.5	0.04 *
	Sex (M/F ratio)	3/28	1/30	0.3 †
	Size (cm)	166±3	167±4	0.3 *
	Weight (kg)	64.7±3.6	65±8	0.4 *
**The performed thyroid surgery**	Total/partial thyroidectomy	14/17	24/7	0.009 ‡
**Intraoperative anesthetic parameters (at 60 minutes)**	HR (bpm)	72.9±8	72.7±11	0.3 *
	SBP (mmHg)	94.7±15	98±14.6	0.4 *
	MAP (mmHg)	70.7±10.4	75.2±10	0.15 *
	DBP (mmHg)	58.6±9.5	63.2±10.2	0.1 *
	SpO _2_ (%)	99.6±0.8	99.7±0.6	0.1 *
	PetCO _2_ (%)	34.6±1.8	34.6±2.9	0.4 *
**Surgery duration**	(minutes)	112±2	107±13	0.09 *

Values are mean±standard deviation.

S: physiological serum, K: ketamine, cm: centimeters, kg: kilograms, HR: heartbeat, bpm: beats per minute, SBP: systolic blood pressure, MAP: mean arterial pressure, DBP: diastolic blood pressure, SpO _2_: oxygen saturation, PetCO _2_: Post apneic end-tidal carbon dioxide pressure.

* Student t-test.

^†^ Fisher exact test.

^‡^ χ ^2^ test.

### Assessment of postoperative pain

Pain scores during swallowing were significantly lower for group K in the PACU at 0, 10, and 20 minutes ( Table 2).

**Table 2.** Mean VAS values in group S and group K at rest and during swallowing in the PACU admission period.

Timing (minutes)	VAS values at rest in the first 2 hours postoperatively			VAS values during swallowing in the first 2 hours postoperatively		
	Group S	Group K	P value	Group S	Group K	P value
0	4.52	1.94	0.05	6.13	2.58	0.04
10	3.87	1.94	0.08	6.45	2.58	0.04
20	3.87	1.94	0.08	6.77	2.58	0.03
30	4.52	2.58	0.12	7.10	3.23	0.05
40	4.84	2.58	0.16	6.13	2.90	0.05
50	4.19	2.58	0.19	6.13	3.23	0.08
60	3.87	2.58	0.19	5.81	3.23	0.09
70	3.87	2.58	0.15	5.81	3.23	0.09
80	4.19	3.23	0.27	6.77	3.87	0.09
90	3.87	3.23	0.33	6.13	3.87	0.1
100	3.87	3.23	0.3	6.13	3.55	0.09
110	4.19	3.23	0.26	6.45	3.55	0.07
120	4.19	3.23	0.26	6.45	3.55	0.07

Group S: saline solution group, group K: ketamine group, VAS: visual analog scale, PACU: post anesthetic care unit.

Regarding the first 24-hour postoperative hospitalization period in the ENT department, the mean VAS values were higher in group S compared to group K; either at rest or during swallowing, but the difference was not statistically significant (p>0.05) ( Table 3).

**Table 3.** Mean VAS values in group S and group K at rest and during swallowing in the first 24 hours postoperatively.

Timing (hours)	VAS values at rest in the first 24 hours postoperatively			VAS values during swallowing in the first 24 hours postoperatively		
	Group S	Group K	P value	Group S	Group K	P value
6	3.87	2.9	0.2	4.52	4.52	0.5
12	3.55	2.9	0.3	4.19	4.19	0.5
18	3.55	2.58	0.2	4.84	4.19	0.3
24	3.55	2.58	0.2	4.84	3.55	0.2

Group S: saline solution group, Group K: ketamine group, VAS: visual analog scale.

None of the patients in group K received morphine while it was administered to four patients in group S. The mean morphine consumption in the PACU was 0.71 mg and 0 mg respectively in group S and group K. The difference in morphine consumption between both groups was statistically significant (p=0.04). Nefopam was not administered to any of our patients.

No patient presented a hematoma at the injection points of the product. A patient in group K showed hallucinations during the stay in the PACU. Dizziness was recorded in three patients of group K and one patient of group S. No patients presented postoperative shivers.

Respiratory distress was not recorded in any of our patients. Nausea and vomiting were observed in a total of 11 patients but without significant differences between both groups.

## Discussion

Acute pain is one of the most common complaints after surgery. Thyroid surgery is known to be responsible for mild to moderate postoperative pain during the first 24 hours, with a mean VAS score of 6.9 (±1.7). ^1^^,^^14^ Pain following thyroid surgery has several origins: surgery-induced inflammatory lesions, intraoperative neck hyperextension, postoperative drainage, incision site, and laryngotracheal mobilization at swallowing. ^15^^,^^16^

The attempted locoregional anesthetic approaches to reduce post-thyroidectomy pain have included mainly bilateral superficial cervical plexus block and local wound infiltration with a local anesthetic agent. ^1^^,^^6^^,^^7^

Wound infiltration with a local anesthetic agent is a simple and safe procedure to reduce postoperative pain with fewer side effects in comparison with cervical plexus block. ^6^^,^^7^ Several molecules were used to infiltrate the thyroidectomy wound. Bupivacaine has been reported to effectively reduce post-thyroidectomy pain. ^10^^,^^16^^,^^17^ Sellami *et al.* ^10^ concluded that infiltration of bupivacaine wounds is effective in reducing pain perception and opioid requirements after thyroidectomy. In a recent meta-analysis, Jiang *et al.* ^17^ recommended performing a local infiltration using bupivacaine before or after skin closure, 20 to 75 mg, as it significantly reduced postoperative pain and decreased rescue analgesic requirements.

Diclofenac has also been reported as an effective molecule to infiltrate the wound before surgery compared to Bupivacaine in reducing postoperative pain. ^11^ However, the use of ropivacaine was not associated with an analgesic effect. ^12^

Ketamine also belongs to the molecules whose local wound analgesic effect has been studied. The local ketamine analgesic effect is related to different mechanisms: blocking sodium, calcium, and potassium channels, as well as binding to opioid, cholinergic, D2, and 5-HTE receptors and monoamine transporters. ^9^^,^^18^^,^^19^ It also decreases microglial activation and migration and prevents local inflammation extension and exacerbation through its action on the prototype inflammatory mediators, the adenosine receptor system, and the activation of the NMDA receptors. ^20^ However, only a limited number of studies demonstrated the analgesic effect of ketamine in thyroid surgery, either locally or systemically.

In our study, ketamine (2 mg/kg) was injected into the wound after skin closure. It was associated with a significant reduction in VAS scores during swallowing compared to the saline solution group in PACU at 0, 10, and 20 minutes. In a prospective, controlled, double-blind, randomized study, Abd EL-Rahman *et al.* ^9^ compared post-thyroidectomy analgesia in three different groups using local ketamine wound instillation, intramuscular ketamine injection, or placebo. He recorded that VAS scores at rest or during movement, as well as morphine consumption, were reduced in the local ketamine group compared to intramuscular ketamine and placebo. He then concluded that local ketamine instillation of 1 mg/kg of ketamine diluted in 10 ml of a saline solution was associated with a superior analgesic effect compared to the other groups. He also recorded that total morphine consumption and first analgesia demand were significantly lower in the local ketamine group.

We also demonstrated that local injection of ketamine significantly reduced the average morphine requirement compared to the saline solution group. The use of opioids can be associated with severe side effects such as nausea, vomiting, and respiratory distress. ^15^ Thus, modern analgesic approaches aim to reduce both postoperative pain and opioid requirements. ^4^^,^^5^

Kim *et al.* ^21^ also studied the effect of intravenous ketamine infusion during the bilateral axilla-breast approach for robotic or endoscopic thyroidectomy and concluded that it significantly reduced postoperative pain scores compared to the saline solution group with no increase in adverse effects. Lee *et al.* ^15^ used ketamine infusion after robotic thyroidectomy and demonstrated that it was associated with a lower VAS score 24 hours after surgery and with a decrease in opioid needs.

The use of ketamine as a local wound infiltration agent was studied more frequently in oral, tonsillar, and abdominal surgeries. In their meta-analysis, Cho *et al.* ^22^ concluded that the use of ketamine locally or systematically could provide pain relief in children undergoing tonsillectomy without side effects. The addition of ketamine to ropivacaine for local tonsillar application was associated with better postoperative analgesia compared to the application of ropivacaine alone. ^23^ Dal *et al.* ^24^ proved that locally injected ketamine effectively reduced pain scores in patients undergoing adenotonsillectomy. However, in a recent prospect guideline for post-tonsillectomy pain management, Aldalmulij *et al.* ^25^ did not recommend the use of ketamine infiltration in children due to the risk of systemic side effects after absorption, although different studies consistently concluded that it was effective in reducing pain and analgesic requirements after tonsillectomy in children.

Infiltrating the surgical site using ketamine in pediatric patients undergoing cleft palate surgery was superior to Bupivacaine with respect to analgesic requirements, quality of sleep after surgery, and speed of recovery. ^26^ In third molar surgery, a meta-analysis based on prospective clinical trials and randomized controlled trials demonstrated the analgesic effect of local administration of ketamine and its anti-inflammatory potential during the first 24 hours. ^27^

Regarding abdominal surgeries, Honarmad *et al.* ^28^ conducted a randomized, double-blind, prospective, placebo-controlled study, and concluded that intravenous or subcutaneous infiltration prior to incision of 0.5 mg/kg of ketamine improved analgesia during the first six hours after appendectomy with no significant side effects. The same authors reported that subcutaneous ketamine infiltration or intravenous ketamine administration for patients undergoing open cholecystectomy before the surgical incision provided auxiliary analgesia 24 hours after surgery.

Our study has several limitations. First, thyroid surgeries were performed by different surgeons. Second, the VSA remains a subjective scale, especially since its assessment during the PACU stay was relatively uneasy with the residual anesthetic effect. We did not consider the size of the resected thyroid gland which can reflect the extent of surgery and influence postoperative pain.

## Conclusions

In conclusion, local wound infiltration using 2 mg/kg of ketamine is an effective approach to reducing opioid requirements after thyroid surgery without increasing the side effects of ketamine.

## Data Availability

Figshare: Underlying data for ‘Ketamine infiltration decreases opioid requirement after thyroid surgery’, https://doi.org/10.6084/m9.figshare.21365703.v1. ^29^ This project contains the following underlying data:
‐ data ketamine thyroidectomy.sav (SPSS format; all datasets have been de-identified in accordance with the Safe Harbor method) ‐ CONSORT checklist Data are available under the terms of the Creative Commons Attribution 4.0 International Public Licence (CC-BY 4.0).
