# Supplementary material for: Ketamine infiltration improves analgesia after thyroid surgery
Source: F1000Res. 2023 Aug 18;12:206. Originally published 2023 Feb 23. [Version 2] doi: 10.12688/f1000research.127562.2 (PMC10835105; doi:10.12688/f1000research.127562.2)
Supplement: Peer review history

# Reviewer report: Reviewer response for version 2

**DOI:** 10.5256/f1000research.154754.r235357

**Published:** 2024-02-01

**Authors:** Pham Quang Minh

**Affiliations:** 1 Hanoi Medical University, Hanoi, Vietnam

1. The study design:

 - Random grouping is unequal: The proportion of patients undergoing total thyroidectomy or partial thyroidectomy is much different between the two groups. Different types of surgery result in different level of pain. It make great influence on research results.

 - The post-operative pain relief design is not appropriate. When VAS score > 30, author used Nefopam. Nefopam is a strong pain relief but it causes a tachycardia so it needs to be infused slowly, with an infusion time is 30 minutes or more. So, this medicine is not suitable for pain relief. An NSAID group such as Ketorolac can be used. Furthermore, when using Nefopam, the dose must also be controlled, not exceeding 120mg per day. So if the patient has already taken 120 mg and still has pain, what is the next treatment?

 - Patients were assessed for pain both at rest or swallowing, but when swallowing, the VAS score was very high in the group not using Ketamine. This is not clinically appropriate in the first 120 minutes, because when author knew the patients were in pain, author still force the patient to perform swallowing for research without giving any additional pain relief. It can be said that author did not respect research ethics.

 2. Data results

 - It need to add a table of results about side effects of pain relief methods, especially hallucinations when using Ketamine. The research object and method section should clearly state: Criteria for diagnosing hallucinations, prevention and treatment.

 ​3. Discussion and conclusion 

 ​​​​​​- Discussion does not focus on the question research or the research results.

Is the work clearly and accurately presented and does it cite the current literature?

Yes

If applicable, is the statistical analysis and its interpretation appropriate?

Partly

Are all the source data underlying the results available to ensure full reproducibility?

Partly

Is the study design appropriate and is the work technically sound?

Partly

Are the conclusions drawn adequately supported by the results?

Yes

Are sufficient details of methods and analysis provided to allow replication by others?

Partly

Reviewer Expertise:

Anesthesiologist

I confirm that I have read this submission and believe that I have an appropriate level of expertise to confirm that it is of an acceptable scientific standard, however I have significant reservations, as outlined above.

# Reviewer report: Reviewer response for version 2

**DOI:** 10.5256/f1000research.154754.r197863

**Published:** 2023-09-04

**Authors:** Chihebeddine Romdhani, Widd Kaabi

**Affiliations:** 1 Faculty of Medicine of Tunis, Universite de Tunis El Manar, Tunis, Tunis, Tunisia; 2 Research Unit UR17DN05, Military Hospital of Tunis, Tunis, Tunisia

The authors have addressed the requested revisions.

Is the work clearly and accurately presented and does it cite the current literature?

Yes

If applicable, is the statistical analysis and its interpretation appropriate?

Yes

Are all the source data underlying the results available to ensure full reproducibility?

Yes

Is the study design appropriate and is the work technically sound?

Yes

Are the conclusions drawn adequately supported by the results?

Yes

Are sufficient details of methods and analysis provided to allow replication by others?

Yes

Reviewer Expertise:

Regional anesthesia, Anesthesiology, critical care, cardiac anesthesia, artificial intelligence

We confirm that we have read this submission and believe that we have an appropriate level of expertise to confirm that it is of an acceptable scientific standard.

# Reviewer report: Reviewer response for version 1

**DOI:** 10.5256/f1000research.140082.r187838

**Published:** 2023-08-10

**Authors:** Mohamed Kahloul

**Affiliations:** 1 Department of Anesthesia and Intensive Care, Sahloul Academic Hospital, Faculty of Medicine of Sousse, University of Sousse, Sousse, Sousse, Tunisia

**Title: **

- Postoperative opioid requirements were assessed only in the PACU. Postoperative pain is your primary outcome. Please rephrase your title.

**Methods**:

- Difficult airways should be considered as an  exclusion criteria because you had assessed VAS during swallowing.
- Please precisely include which ketamine concentration was used.

**Results:**

- Baseline characteristics should be comparable between the two groups. Please verify your data (age, performed surgery).
- You have many VAS assessments exceeding 3/10. What was your rescue analgesia?

**Discussion:**

- The choice of ketamine dosage should be discussed.
- "However, the use of ropivacaine was not associated with an analgesic effect. ^12"^ Others studies had found different results and they should be considered ^1^.
- VAS was considered as a limitation while it is a validated tool.

**Conclusion:**

- Please put “to reduce” instead of “to reducing”.

Is the work clearly and accurately presented and does it cite the current literature?

Yes

If applicable, is the statistical analysis and its interpretation appropriate?

Partly

Are all the source data underlying the results available to ensure full reproducibility?

Partly

Is the study design appropriate and is the work technically sound?

Yes

Are the conclusions drawn adequately supported by the results?

Yes

Are sufficient details of methods and analysis provided to allow replication by others?

Yes

Reviewer Expertise:

Anesthesia and intensive care

I confirm that I have read this submission and believe that I have an appropriate level of expertise to confirm that it is of an acceptable scientific standard, however I have significant reservations, as outlined above.

## References

[1] : Benefits of ropivacaine infiltration for reducing postoperative pain after thyroid surgery: a meta-analysis of randomized controlled trials. *Int J Clin Exp Med* .2019;12(6) : Reference source

# Response

**Published:** 2023-08-17

**Authors:** Moncef Sellami

**Affiliations:** Habib Bourguiba University Hospital, Tunisia

**We express our gratitude to the reviewer for his meticulous assessment, which has contributed significantly to the refinement of our study.**

 Title:

- Postoperative opioid requirements were assessed only in the PACU. Postoperative pain is your primary outcome. Please rephrase your title.

**Author response: The title has been revised as 'Ketamine infiltration improves analgesia after thyroid surgery'.**

 Methods:

- Difficult airways should be considered as an  exclusion criteria because you had assessed VAS during swallowing.
- Please precisely include which ketamine concentration was used.

**Author response: **

- **In effect, difficult airways were considered an exclusion criterion.**
- **We specified the ketamine concentration used, which was 50mg/ml**

Results:

- Baseline characteristics should be comparable between the two groups. Please verify your data (age, performed surgery).
- You have many VAS assessments exceeding 3/10. What was your rescue analgesia?

**Author response:**

- **The patients were randomized and the results were statistically verified.**
- **Our data, including age and type of surgery, have been carefully validated.**
- **We specified that the rescue analgesic during hospitalization was Nefopam in the case of VAS values greater than 30**

Discussion :

- The choice of ketamine dosage should be discussed.

**Author response:**

**In our study, we infiltrated a solution containing 2 mg/kg ketamine based on established literature and clinical experience, in order to achieve effective analgesia while minimizing potential adverse effects.**

**We discuss the chosen dose.**

- "However, the use of ropivacaine was not associated with an analgesic effect. ^12^ ^"^ Others studies had found different results and they should be considered ^1^ .

**Author response:**

**We discussed this issue and reported the study by Li X et al.**

  

- VAS was considered as a limitation while it is a validated tool.

**Author response:**

**We have considered the reviewer's comment and revised the limitations section of the study. Indeed, the VAS is a validated tool despite its subjective nature.**

 Conclusion:

- Please put “to reduce” instead of “to reducing”.

**Author response:**

**We have made the requested change, replacing "to reducing" with "to reduce" in the relevant section.**

# Reviewer report: Reviewer response for version 1

**DOI:** 10.5256/f1000research.140082.r187834

**Published:** 2023-07-31

**Authors:** Chihebeddine Romdhani, Widd Kaabi

**Affiliations:** 1 Faculty of Medicine of Tunis, Universite de Tunis El Manar, Tunis, Tunis, Tunisia; 2 Research Unit UR17DN05, Military Hospital of Tunis, Tunis, Tunisia

In this study, researchers investigated the effects of local ketamine wound infiltration versus saline placebo on postoperative pain and opioid consumption in patients undergoing thyroid surgery. After randomizing 64 patients to receive either ketamine or saline infiltration, the researchers found that the ketamine group had lower VAS pain scores during the PACU stay for static pain at rest and dynamic pain on swallowing. The ketamine group consumed significantly less morphine in the PACU than the saline group. No adverse effects were reported. The authors concluded that local ketamine can reduce postoperative opioid use without increasing side effects. This study is well-designed and addresses an important clinical question.

 We recommend you to :

- Provide more details regarding the randomization process: Was a computer random number generator used? Were block randomization or stratification techniques used?
- Presenting VAS scores graphically could better visualize trends.

Is the work clearly and accurately presented and does it cite the current literature?

Yes

If applicable, is the statistical analysis and its interpretation appropriate?

Yes

Are all the source data underlying the results available to ensure full reproducibility?

Yes

Is the study design appropriate and is the work technically sound?

Yes

Are the conclusions drawn adequately supported by the results?

Yes

Are sufficient details of methods and analysis provided to allow replication by others?

Yes

Reviewer Expertise:

Regional anesthesia, Anesthesiology, critical care, cardiac anesthesia, artificial intelligence

We confirm that we have read this submission and believe that we have an appropriate level of expertise to confirm that it is of an acceptable scientific standard.

# Response

**Published:** 2023-08-17

**Authors:** Moncef Sellami

**Affiliations:** Habib Bourguiba University Hospital, Tunisia

**We thank reviewers for the quality and relevance of the feedback.**

 In this study, researchers investigated the effects of local ketamine wound infiltration versus saline placebo on postoperative pain and opioid consumption in patients undergoing thyroid surgery. After randomizing 64 patients to receive either ketamine or saline infiltration, the researchers found that the ketamine group had lower VAS pain scores during the PACU stay for static pain at rest and dynamic pain on swallowing. The ketamine group consumed significantly less morphine in the PACU than the saline group. No adverse effects were reported. The authors concluded that local ketamine can reduce postoperative opioid use without increasing side effects. This study is well-designed and addresses an important clinical question.

 We recommend you to :

 Provide more details regarding the randomization process: Was a computer random number generator used? Were block randomization or stratification techniques used?

**Author response:**

**The patients were randomly assigned to the two groups through an online random list generator (https://www.random.org), ensuring an unbiased allocation. The randomization method was specified.**

 Presenting VAS scores graphically could better visualize trends.

**Author response:**

**Although we appreciate the suggestion to present VAS scores graphically, we found that tabulated data provide a more precise and interpretable representation in this particular context.**
